# Stemness-related LncRNA pair signature for predicting therapy response in gastric cancer

**DOI:** 10.1186/s12885-021-08798-1

**Published:** 2021-09-29

**Authors:** Quan Jiang, Hao Chen, Zhaoqing Tang, Jie Sun, Yuanyuan Ruan, Fenglin Liu, Yihong Sun

**Affiliations:** 1 Department of General Surgery, Zhongshan Hospital, Fudan University, Shanghai, 200032 China; 2 Human Phenome Institute, Fudan University, Shanghai, 200433 China; 3 Department of Biochemistry and Molecular Biology, School of Basic Medical Sciences, Fudan University, Shanghai, 200032 China

## Abstract

**Objective:**

As a critical feature of cancers, stemness is acknowledged as a contributor to the development of drug resistance in gastric cancer (GC). LncRNAs have been revealed to participate in this process. In this study, we tried to develop a stemness-related lncRNA pair signature as guidance for clinical decisions.

**Methods:**

The analysis was initiated by collecting stemness-related lncRNAs in TCGA cohort. The differentially expressed stemness-related lncRNAs between normal and tumor tissues in GC patients from TCGA datasets were further collected to establish the signature based on Lasso and Cox regression analyses. The predictive efficacy of the signature for chemotherapy and immunotherapy was also tested. The practicality of this signature was also validated by Zhongshan cohort.

**Results:**

A 13-DEsrlncRNA pair-based signature was established. The cutoff point acquired by the AIC algorithm divided the TCGA cohort into high and low risk groups. We found that the low-risk group presented with better survival (Kaplan-Meier analysis, *p* < 0.001). Cox regression analyse was also conducted to confirm the signature as an independent risk factor for GC {p < 0.001, HR = 1.300, 95% CI (1.231–1.373)]}. As for the practicality of this signature, the IC50 of cytotoxic chemotherapeutics was significantly higher in the high-risk group. The low-risk group also presented with higher immunophenoscore (IPS) in both the “CTLA4+ PD1+” (Mann-Whitney U test, *p* = 0.019) and “CTLA4- PD1+” (Mann-Whitney U test, *p* = 0.013) groups, indicating higher sensitivity to immunotherapy. The efficacy of the signature was also validated by Zhongshan cohort.

**Conclusions:**

This study could not only provide a stemness-related lncRNA signature for survival prediction in GC patients but also established a model with predictive potentials for GC patients’ sensitivity to chemotherapy and immunotherapy.

**Supplementary Information:**

The online version contains supplementary material available at 10.1186/s12885-021-08798-1.

## Introduction

As a heterogeneous disease with a worldwide presence, GC is ranked as the fifth most common malignant tumor and the fourth leading cause of cancer-related mortality worldwide [1, 2]. Despite all the efforts devoted to improving the curative effect in the last decade, the treatment outcomes of GC remain unsatisfactory. As concluded in previous studies, more than two-thirds of GC cases are diagnosed at advanced stages globally [3]. Compared with the treatment outcomes in western countries, GC patents in eastern Asia are diagnosed at a relatively early stage and thus get better survival. However, there is still much room for improvement in treatment outcomes since the median overall survival is only 11 months with combination chemotherapy [4]. Later lines of systemic therapy, such as ramucirumab and trifluridine/tipiracil, improve survival by only 1 to 2 months compared with placebo. New effective therapeutics are needed [5, 6].

Immune checkpoint blockade (ICB) has brought about unprecedented hope for cancer patients losing the opportunity for radical surgery, yet the clinical benefits are still limited. For the ICB treatments of GC, the current circumstances are not satisfactory. In 2017, a programmed cell death–1 (PD-1) inhibitor named pembrolizumab was granted accelerated approval from the US Food and Drug Administration for the treatment of recurrent locally advanced or metastatic gastric cancer. Approval was made based on a single-armed phase 2 clinical trial [7]. Previous studies hold the opinion that tumors with higher expression levels of programmed cell death ligand 1 (PD-L1) are more likely to benefit from PD-1 inhibitors. However, the objective response rate in PD-L1– high tumors was finally revealed to be 11.6 and 11.2% in two independent PD-1 inhibitor-concerning clinical trials [8, 9]. The development of a model for the proper selection of GC patients who can benefit from ICB therapy is urgently needed.

Stemness has long been recognized as one of the most important characteristics of tumor cells. It is acknowledged that stemness may lead to tumor recurrence and chemotherapy resistance. However, it remains unknown whether stemness may affect the efficacy of immunotherapy in GC. Defined as transcripts that are longer than 200 nt, long noncoding RNAs (lncRNAs) have been identified as crucial factors affecting tumor biological function. The aberrant expression of lncRNAs has been revealed to have a profound impact on cancer biological behaviors such as proliferation, progression and metastasis [10]. The correlations between lncRNAs and cancer stemness cells (CSCs) have been revealed in previous studies [10]. Many lncRNAs have been reported to affect the biological functions of cancer by regulating the stemness of cancer cells. It has been reported that DANCR could enhance the stemness characteristics of hepatocellular carcinoma by lowering the expression of CTNNB1, which thus leads to chemotherapy resistance [11]. Moreover, the development of cancer stem cells (CSCs) in breast cancers was found to be highly linked with a novel lncRNA named lnc030, which may stabilize SQLE mRNA and increase stemness features [12]. Recent evidence has also suggested that lncRNAs contribute to the malignant phenotypes of cancer not only through genomic or transcriptomic alterations but also by altering the immune microenvironment [13]. However, the stemness related lncRNAs have still not been well elucidated in GC and the interactions among them were also obscure. Despite that multiple lncRNAs have been revealed to participate in the development of stemness feature, the most crucial lncRNAs also remained unknown. Considering that lncRNAs were highly correlated to exert similar biological functions, it is urgently needed to find out the potential mechanisms hidden behind.

In this study, we aimed to develop a stemness-related lncRNA pair signature with guiding significance for clinical decisions. This study could not only provide a stemness-related lncRNA signature for survival prediction in GC patients but also established a model with predictive potentials for GC patients’ sensitivity to chemotherapy and immunotherapy.

## Materials and methods

### Data source and DEsrlncRNA collection

The RNA-seq data and clinical profiles of GC patients were derived from the TCGA Data Portal (https://portal.gdc.cancer.gov/, December 10, 2020). A total of 375 patients who had integral lncRNA and mRNA expression profiles, survival information and common clinicopathological characteristics were enrolled in this signature establishment process. GTF files were downloaded from Ensembl (http://asia.ensembl.org) for annotation to distinguish the mRNAs and lncRNAs. RNA-seq and survival data of 29 GC patients enrolled in clinical trial concerning neoadjuvant chemotherapy were also acquired. Mandard tumor regression grade (TRG) was applied as the criteria for calculating the chemotherapy response of patients [14]. This system classifies pathologic response as follows: TRG 1 (complete regression/fibrosis with no evidence of tumor cells), TRG 2 (fibrosis with scattered tumor cells), TRG 3 (fibrosis and tumor cells with a dominance of fibrosis), TRG 4 (fibrosis and tumor cells with a dominance of tumor cells), and TRG 5 (tumor without evidence of regression). Grade 1–2 was defined as major response while Grade 3–5 as minor response. A list of stemness-related genes (SRGs) was acquired from a previously published review [15] and was used to screen stemness-related lncRNAs by a coexpression strategy under the threshold of coefficient value > 0.4 and *p* value < 0.05. To further collect the differentially expressed stemness-related lncRNAs (DEsrlncRNAs), differential expression analysis between normal (*N* = 32) and tumor (*N* = 375) RNA-seq data was conducted with the R package “limma” under the threshold of |fold change (FC) > 1| and false discovery rate (FDR) < 0.01.

### Pairing DEsrlncRNAs

The DEsrlncRNAs were cyclically singly paired, and a 0-or-1 matrix was constructed assuming C was equal to lncRNA A plus lncRNA B; C was defined as 1 if the expression level of lncRNA A was higher than that of lncRNA B; otherwise, C was defined as 0. Then, the constructed 0 or 1 matrix was further screened. No relationship was considered between pairs and prognosis if the expression quantity of lncRNA pairs was 0 or 1 because pairs without a certain rank could not properly predict the patient survival outcome. When the number of lncRNA pairs whose expression quantity was 0 or 1 accounted for more than 20% but less than 80% of the total pairs, it was considered a valid match. The process was originally and specifically described in previous publications [16].

### Construction of the DEsrlncRNA pair-based Riskscore

Lasso regression was performed with 10-fold cross validation and a *p* value of 0.05 based on the results of the univariate analysis for DEsrlncRNA pairs. A total of 1000 cycles were run, and random stimulation was performed 1000 times in each cycle. Next, the frequency of each pair was acquired, and pairs with frequencies greater than 100 were collected for further Cox regression analysis. The pairs with independent prognostic predictive value were finally enrolled for the construction of the Riskscore model with the following formula:

$$ Riskscore={\sum}_{i=1}^n coef\left( lncRNAi\ pair\right)\ast expr\left( lncRNAi\ pair\right) $$

In this formula, *lncRNA pair* represents the prognosis-related lncRNA pairs derived from Cox regression analysis. *Expr (lncRNAi pair)* indicates the value of the lncRNAi pair (1 or 0). *Coef (lncRNAi pair)* is the correlation coefficient of the lncRNAi pair in the Riskscore model. The AUC value of each model was also calculated from ROC curves. The highest point of the drawn curve represented the maximum AUC value. Different time limitations (1-, 2-, and 3-years) for the ROC curves were all tested. The 1-year ROC curve was applied to evaluate the AIC values to identify the maximum inflection point, which was regarded as the cutoff point to separate the high-risk group from the low-risk group in the established Riskscore model.

Kaplan–Meier curves were drawn to compare the survival difference between the high and low-risk groups with a threshold of *p*-value≤5%. The Wilcoxon signed-rank test was applied to explore the correlations between the Riskscore model and clinicopathological characteristics. The Riskscore model was further tested by Cox regression analysis to assess whether it is an independent prognostic factor for GC patients.

### Evaluation of the significance of the model in Chemosensitivity prediction

To explore the sensitivity of different chemotherapeutic agents for GC patients, the “pRRophetic” package in R software (3.6.1) was applied to obtain the IC50 values of GC-related chemotherapeutics and targeted drugs. This algorithm was previously published and has been widely used in multiple studies [17–19]. Mann-Whitney U test was applied to compare the IC50 values between the low and high-risk groups.

### Functional enrichment analysis

The differentially expressed genes (DEGs) between the low and high-risk groups were acquired with the “limma” package in R software with a threshold of *p* value < 0.05. Gene Ontology (GO) enrichment analysis was conducted based on the DEGs. Kyoto Encyclopedia of Genes and Genomes (KEGG) analysis for two risk stratifications were also performed based on the “cp.kegg.v7.4.symbols.gmt” reference package in GSEA software.

### Exploration of immune-related characteristics and tumor-infiltrating immune cells

Tumor mutation burden (TMB) was defined as the total amount of coding errors of somatic genes, base substitutions, and insertions or deletions detected across per million bases. The TMB score was acquired by calculating the mutation frequency with the number of variants/the length of exons (38 million) for each sample via Perl scripts based on the JAVA8 platform. Spearman correlation analysis was further conducted to analyze the correlations between the Riskscore and the TMB. A total of 17 immune checkpoints (IDO1, CD274, HAVCR2, PDCD1, CTLA4, LAG3, CD8A, CXCL10, CXCL9, GZMA, GZMB, PRF1, IFNG, TBX2, TNF, CD80, and CD86) with potential therapeutic values were also compared between the two risk stratifications.

To determine the relationship between the Riskscore and the tumor immune microenvironment, the currently well-known methods, including TIMER, XCELL, QUANTISEQ, MCP-counter, EPIC, and CIBERSORT, were applied to calculate the proportions of infiltrating immune cells. Spearman correlation analysis was further conducted to analyze the correlations between the Riskscore and the immune cells. The correlation coefficients of the results are shown in a lollipop diagram under the threshold of R > 0.1 and *p*-value < 0.05.

### Investigation of the significance of the model for immune checkpoint blockade

The immunophenoscore (IPS) refers to the four main parts (effector cells, immunosuppressive cells, MHC molecules and immunomodulators) determining immunogenicity, and it is calculated without bias using machine learning methods. The IPS (range 0 to 10) was calculated based on the gene expression in representative cell types. The IPS results of STAD patients were downloaded from The Cancer Immunome Atlas (TCIA) (https://tcia.at/home). The online tool Tumor Immune Dysfunction and Exclusion (TIDE) (http://tide.dfci.harvard.edu) was used to predict the immunotherapeutic responses of each sample based on the transcriptome profiles. The TIDE score was compared between the high and low ICI score groups. A lower TIDE score indicated a relatively better response to immunotherapy.

### RNA extraction and sequencing

Formalin fixation and paraffin embedding tissues of 29 patients enrolled in clinical trial concerning neoadjuvant chemotherapy were acquired from the pathology department of Zhongshan Hospital after obtaining the patient’s informed consents. Total RNA was extracted from recently cut 10 mm FFPE sections using the miRNeasy FFPE kit (Qiagen, Valencia, CA) according to the manufacturer’s protocol, using 1–4 sections (10–40 mm) per case depending on assay. RNA yield and quality were determined by UV absorption on a NanoDrop 1000 spectrophotometer and fragment size was analyzed using the RNA 6000 Nano assay (Agilent Technologies, Santa Clara, CA) run on the 2100 Bioanalyzer. DV200 values representing the percentage of RNA fragments above 200 nucleotides in length were determined according to Agilent and Illumina recommended protocols. To determine the minimal amount of tissue needed to yield adequate RNA quantity for library preparation, RNA yield per 10 mm section number was tested. Based on the test results, one or two 10 mm sections of breast FFPE specimens were used for RNA isolation. RNA quality was assessed using DV200 values and cases with DV200 more than 27% were included for library preparation. After Library preparation, Sequencing was performed on a Hi-Seq 2500 using a 100 cycle, single read protocol with a depth of approximately 90 million reads per sample. Following initial sequencing, 3 of the 6 libraries were repooled and independently sequenced. Base call files were converted to fastq format using Bcl2Fastq (Illumina). All RNA-seq reads were aligned to the human reference genome (GRCh38, release 84) using STAR (version 2.5.2b). The studies were reviewed and approved by Ethics Committee of Zhongshan Hospital Affiliated to Fudan University (Approval No: B2017–003).

## Results

### Identification of DEsrlncRNAs and DEsrlncRNA pair construction

**Fig. 1:**
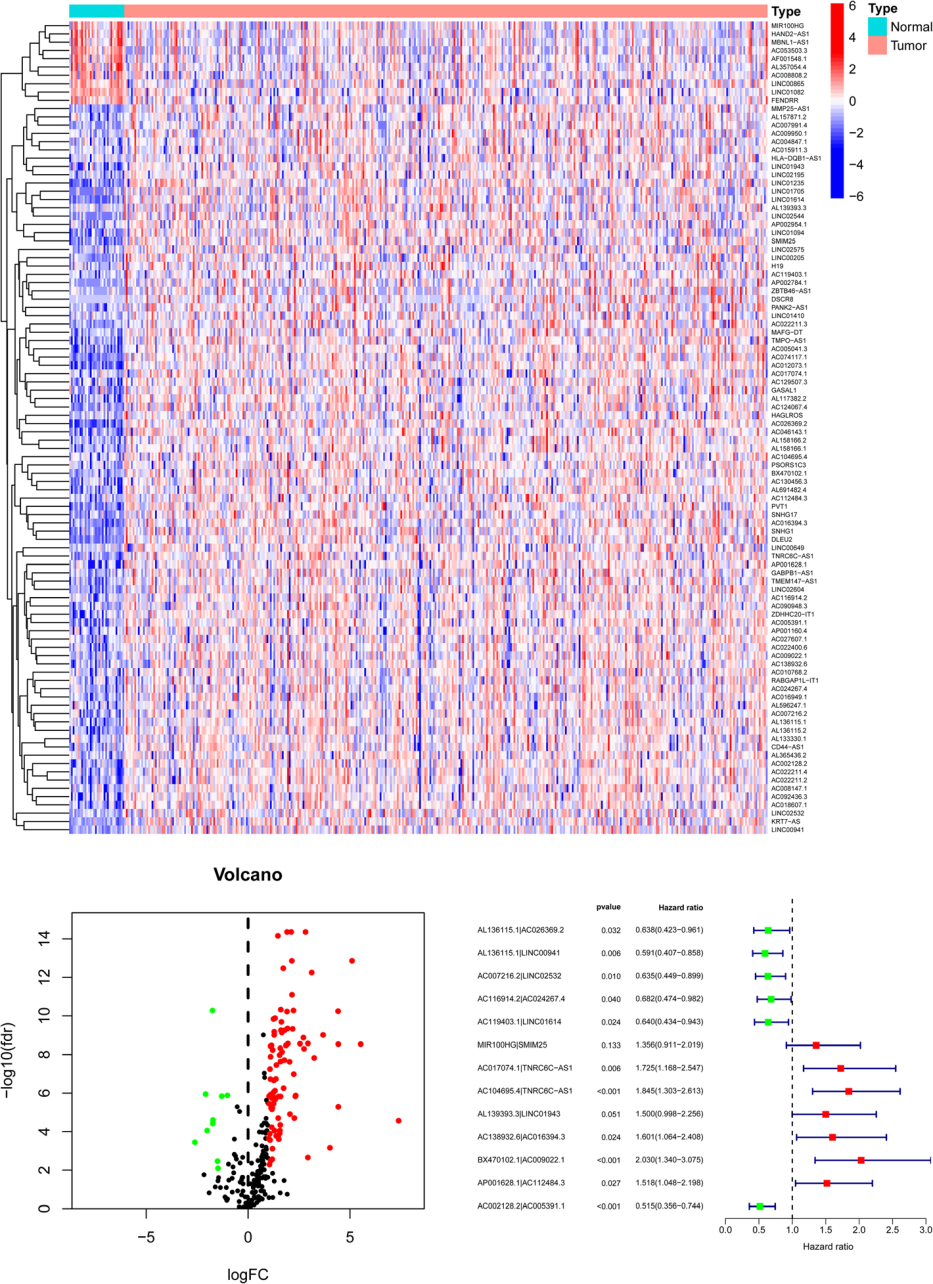
Establishment of the risk assessment model based on DEsrlncRNA pairs. Identification of differentially expressed stemness-related lncRNAs (DEsrlncRNAs) using TCGA STAD datasets. **A** and **B** The lncRNAs are shown by the heatmap (**A**) and volcano plot (**B**). **C** A forest map showing 13 DEsrlncRNA pairs identified by multivariate Cox proportional hazards regression with the stepwise method

The workflow of this study is shown in sFigure 1. The RNA-seq data of the TCGA STAD cohort were firstly applied to annotate and collect the lncRNAs according to gene transfer format (GTF) files from Ensembl. The expression level of 42 SRGs were also extracted from the TCGA STAD RNA-seq data (Supplementary Table 1). To find out the roles SRGs played in GC, we make comparison of them between tumor and normal tissues in TCGA cohort. Besides, we also conducted univariate Cox regression analysis of 42 genes. The results revealed that most of the SRGs were differentially expressed and most of them were found increased in tumor tissues except for KIT, NGFR, SOX2 and KLF4 (sFigure 2A). Furthermore, more than half of SRGs indicated prognostic values based on univariate Cox regression analysis (sFigure 2B). The results suggested that the SRGs might be important for GC development. After that, differentially expressed LncRNAs were secondarily acquired based on the comparisons between 32 normal and 375 tumor RNA-seq data points (*p* ≤ 0.01, log2 fold change = 1). A total of 240 lncRNAs were acquired (Fig. 1A) (Supplementary Table 2). Coexpression analysis was further conducted between stemness-related genes and 240 differentially expressed lncRNAs (Spearman correlation analysis, *p* ≤ 0.05). A total of 98 lncRNAs (88 upregulated and 10 downregulated) were identified as DEsrlncRNAs. The DEsrlncRNAs are presented in a volcano plot (Fig. 1B) (Supplementary Table 3). The 98 lncRNAs were further processed to obtain 13 lncRNA pairs for further establishment of the Riskscore model based on Cox and Lasso regression algorithms (Fig. 1C).

The process of pairing the lncRNAs is described below. Using an iteration loop and a 0-or-1 matrix screening among 98 DEsrlncRNAs, 3231 valid DEirlncRNA pairs were identified. The 3231 pairs were first processed with a univariate Cox regression analysis preliminarily to extract 57 pairs with prognosis-predictive values. After a single factor test followed by Lasso regression analysis, 26 DEsrlncRNA pairs were further selected from the 57 pairs (sFigure 3A and B). Afterwards, univariate Cox regression analysis by the stepwise method was conducted to finally select 13 pairs out of the 26 pairs (sFigure 3C). The 13 pairs were confirmed as independent prognostic factors by a further multivariate Cox regression analysis (Fig. 1C).

### Development of the DEsrlncRNA pair-based Riskscore

**Fig. 2:**
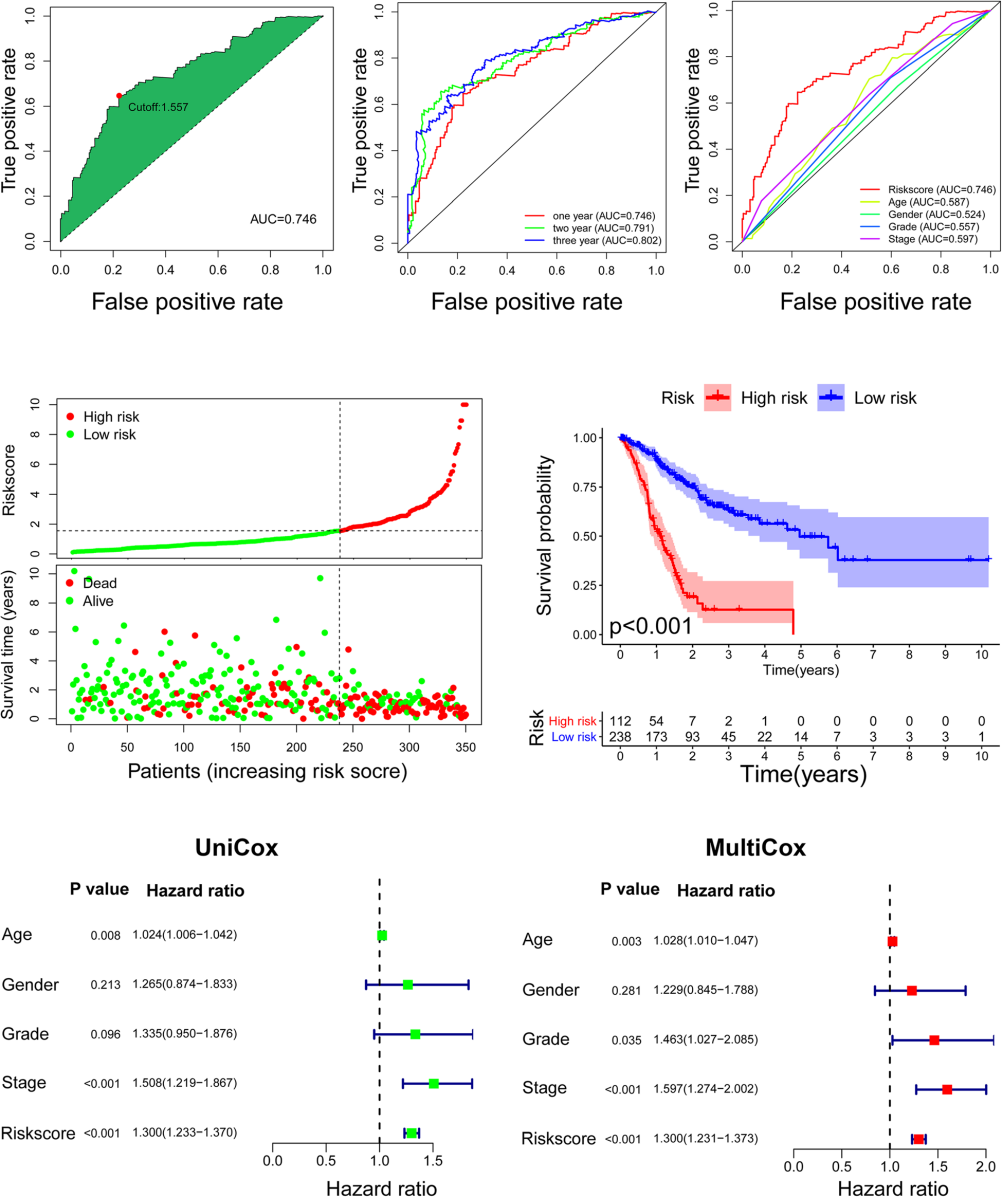
Validations of the prognostic predictive value of the established DEsrlncRNA pair-based signature. **A** The maximum inflection point was the cutoff point obtained by the Akaike information criterion (AIC). **B** The 1-, 2-, and 3-year ROC curves of the model were drawn and revealed that the AUC values were all over 0.7. **C** A comparison of 1-year ROC curves with other common clinical characteristics shows the superiority of the Riskscore. **D** The Riskscores and survival state of all patients are shown. **E** Patients in the low-risk group had a longer survival time, as determined by the Kaplan-Meier method. **F** and **G** Univariate and multivariate Cox analyses confirmed the signature as an independent risk factor for the TCGA STAD cohort

Next, we calculated the areas under the curve (AUCs) for each receiver operating characteristic (ROC) curve of the 13 pairs, drew the curved line, and found the highest point referring to 0.746 for the maximum AUC value (Fig. 2A). The maximum inflection point is also the cutoff point (risk value = 1.557) obtained by the Akaike information criterion (AIC). To validate the optimality, the 1-, 2-, and 3-year ROC curves of the model were also drawn. The corresponding AUC values were 0.746, 0.791 and 0.802 (Fig. 2B). The comparison of the AUC value between the 1-year ROC curve and other clinical characteristics showed the superiority of the Riskscore (Fig. 2C). The survival state of each patient is also presented with scatter plots, and the cutoff point separated the low-risk group (*N* = 238) from the high-risk group (*N* = 112) (Fig. 2D). Patients in the low-risk group experienced better survival based on the Kaplan-Meier test (*p* < 0.0001) (Fig. 2E). The univariate and multivariate Cox regression analyses finally confirmed the signature as an independent risk factor for GC (*p* < 0.001, HR = 1.300, 95% CI [1.231–1.373]) (Fig. 2F and G). Consistently, grade (*p* = 0.034, HR = 1.463, 95% CI [1.027–2.085]) and stage (*p* < 0.001, HR = 1.597, 95% CI [1.274–2.002]) were also revealed as independent risk factors (Fig. 2F and G).

### Analysis of the correlations between the Riskscore and chemotherapeutics and targeted drugs

**Fig. 3:**
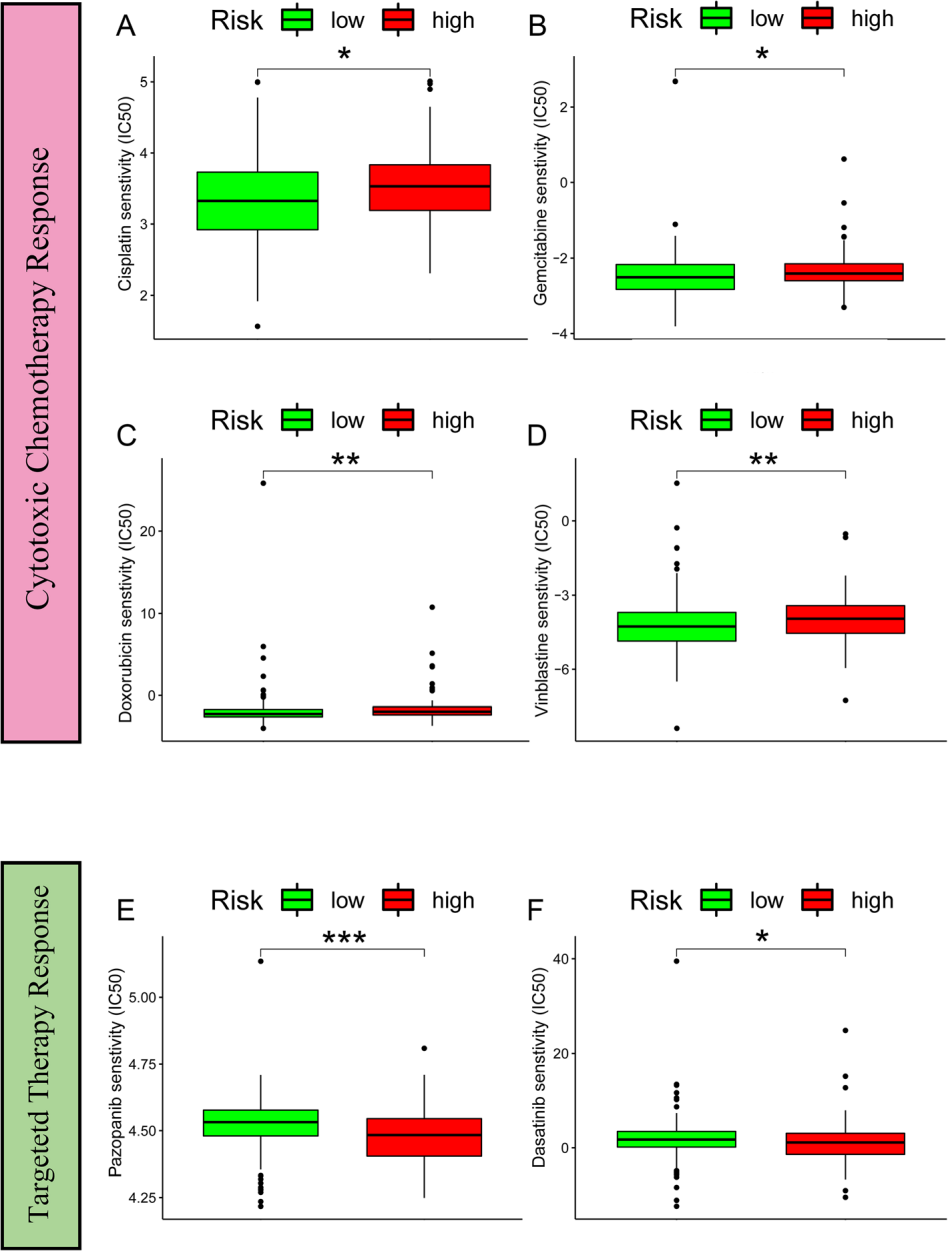
IC50 values for cytotoxic chemotherapeutics between different risk stratifications. **A**-**D** The IC50 values of 4 cytotoxic chemotherapeutics acquired from pRRophetic algorithm, **A** cisplatin, **B** gemcitabine, **C** doxorubicin, and **D** vinblastine, were significantly lower in the low-risk group. **E** and **F** The IC50 values of 2 targeted drugs acquired from pRRophetic algorithm, including **E** pazopanib and **F** dasatinib, were significantly lower in the high-risk group. (Mann-Whitney U test * for *p* < 0.05, ** for *p* < 0.01, and *** for *p* < 0.001)

Stemness has been acknowledged as a predictor of worse response to chemotherapy. Based on the “pRRophetic” package from R software, the IC50 values of 6 GC-related chemotherapeutics and targeted drugs (cisplatin, doxorubicin, gemcitabine, vinblastine, pazopanib, and dasatinib) were calculated for each case. The results revealed that the IC50 values for cytotoxic chemotherapeutics [cisplatin (*p* = 0.018) (Fig. 3A), gemcitabine (*p* = 0.02) (Fig. 3B), doxorubicin (*p* = 0.0031) (Fig. 3C), and vinblastine (*p* = 0.0021) (Fig. 3D)] were significantly higher in the high-risk group. Moreover, the IC50 values for the targeted drugs {pazopanib (*p* < 0.0001) (Fig. 3E) and dasatinib (*p* = 0.03) (Fig. 3F)} were significantly higher in the low-risk group.

### Functional enrichment analysis

**Fig. 4:**
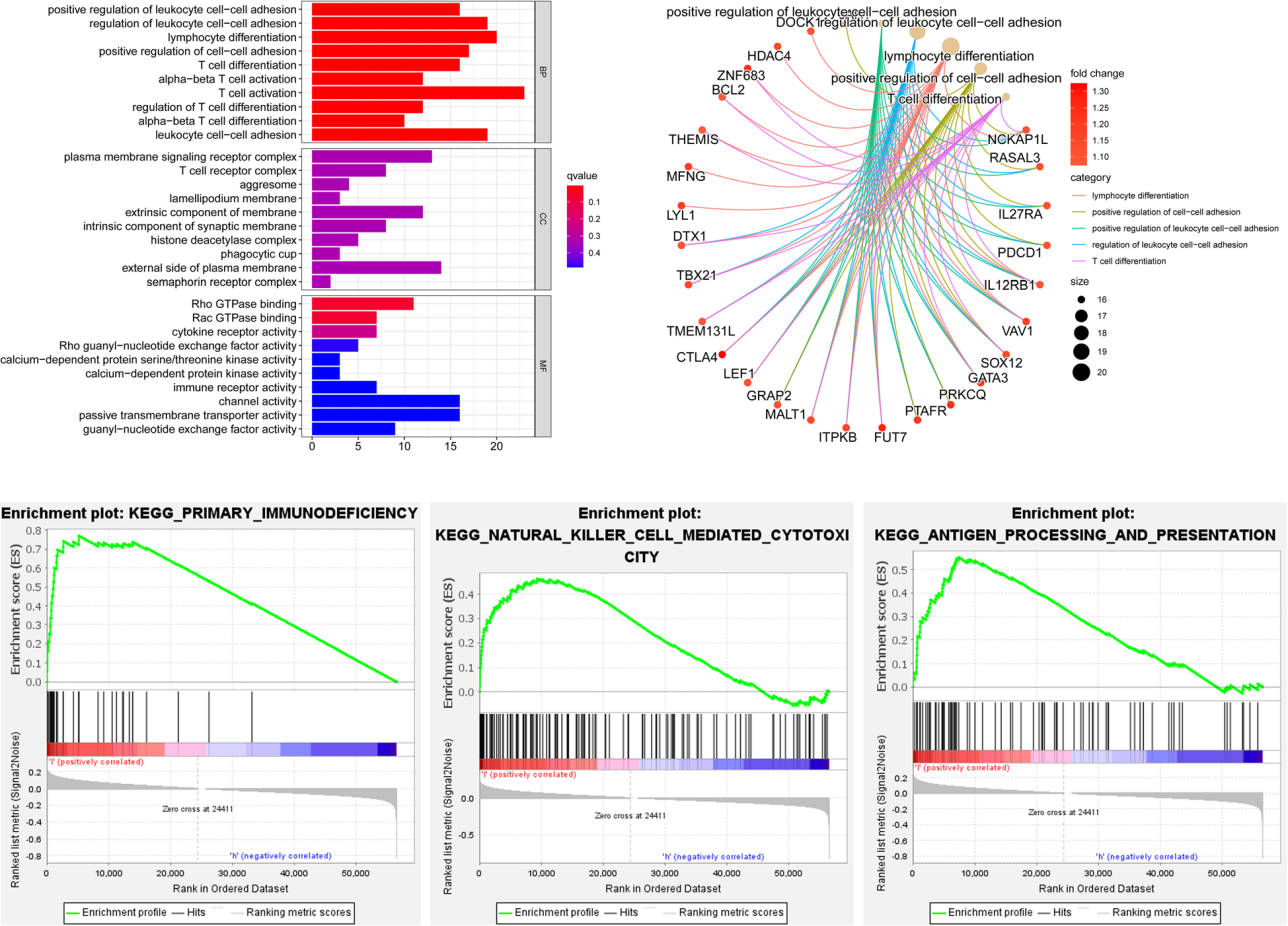
Functional enrichment analysis. **A** Gene Ontology enrichment analysis based on differentially expressed genes (Risk-L vs. Risk-H) indicated that immune-related pathways were highly enriched in low-risk groups. **B** The top 5 pathways and corresponding genes are described in detail by the correlation Circos plot. **C** GSEA Kyoto Encyclopedia of Genes and Genomes (KEGG) analysis based on differentially expressed genes (Risk-L vs. Risk-H) also revealed that the immune-related pathways were highly enriched in low-risk group

Differentially expressed gene (DEG) analysis between the low- and high-risk groups was performed, and 760 DEGs were acquired (Supplementary Table 4). The DEGs were further processed with ID transformation to conduct GO enrichment analysis. The top 5 enriched pathways of the low-risk group were all immune-related, namely “positive regulation of leukocyte cell-cell adhesion”, “regulation of leukocyte cell-cell adhesion”, “lymphocyte differentiation”, “positive regulation of cell-cell adhesion”, and “T cell differentiation” (q value filter< 0.05) (Fig. 4A). The 5 immune-related pathways may suggest that the tumors with lower Riskscore were more likely to be immune-activated since the 5 pathways were all related to immune activation. Furthermore, the top 5 enriched pathways and corresponding genes are described in detail by correlation Circos plots (Fig. 4B).

GSEA KEGG analysis was also conducted. The results revealed that the low-risk group was also highly enriched in immune-related pathways, such as “KEGG_PRIMARY_IMMUNODEFICIENCY” (NES = 1.86, *P* = 0.006), “KEGG_NATURAL_KILLER_CELL_MEDIATED_CYTOTOXICITY” (NES = 1.63, *P* = 0.036) and “KEGG_ANTIGEN_PROCESSING_AND_PRESENTATION” (NES = 1.69, *P* = 0.043) (Fig. 4C). The results showed consistency with to the GO enrichment analysis results above.

### Immune-related characteristics and ICB therapy response based on the DEsrlncRNA pair-based Riskscore

**Fig. 5:**
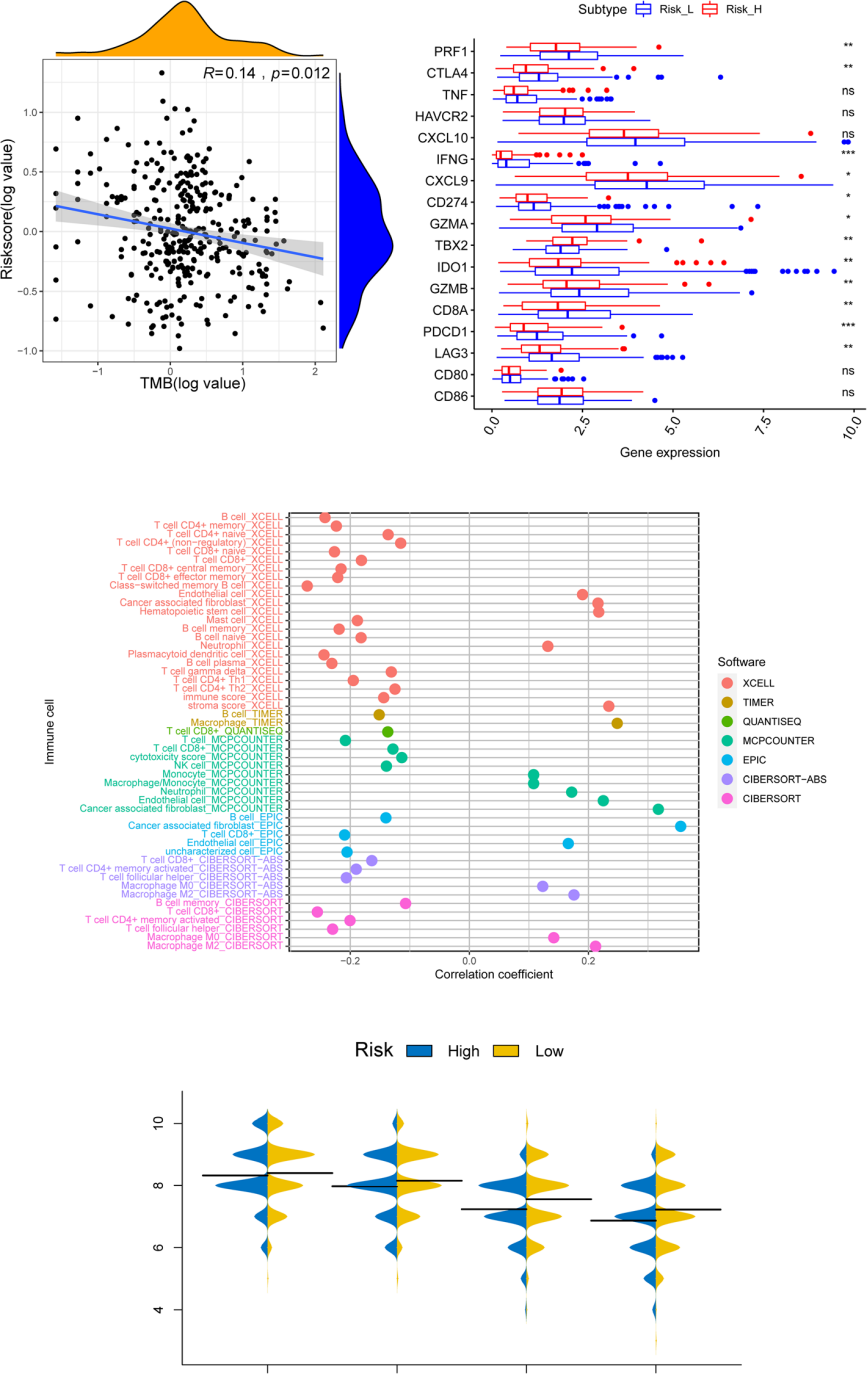
Correlations between the DEsrlncRNA pair-based signature and immunotherapy. **A** Tumor mutation burden was negatively correlated with the Riskscore. **B** The comparisons of 17 immune checkpoints between the two risk groups revealed that most of the markers were higher in the low-risk group. (Mann-Whitney U test’s t test, * for *p* < 0.05, ** for *p* < 0.01, and *** for *p* < 0.001 and ns for not significant). **C** Correlation analysis revealed that immune components were more enriched in the low-risk group (Spearman correlation analysis, *p* ≤ 0.05). (D) IPS analysis revealed that the Riskscore may present a better performance in predicting PD1 blockade therapy than CTLA4 blockade therapy. {“ctla4+ pd1+” (Mann-Whitney U test, *p* = 0.019) and “ctla4- pd1+” (Mann-Whitney U test, *p* = 0.013)}

The functional enrichment analysis above suggested that the Riskscore was obviously related to tumor immunity. Tumor mutation burden (TMB) and the expression level of immune checkpoints are well known as important indicators for the application of ICBs. The Riskscore presented negative correlations with TMB values based on the Spearman correlation analysis (R = -0.14, *p* value = 0.012) (Fig. 5A). Since TMB is recognized as a better treatment response predictor of ICB, the lower value of the Riskscore was again shown to have similar potential. The expression levels of 17 immune checkpoints, including IDO1, CD274, HAVCR2, PDCD1, CTLA4, LAG3, CD8A, CXCL10, CXCL9, GZMA, GZMB, PRF1, IFNG, TBX2, TNF, CD80, and CD86, were collected from the RNA-seq data. The comparisons between the two risk groups revealed that most of the markers presented with significant differences in expression levels (Kruskal-Wallis test) (Fig. 5B). Of all the differentially expressed immune checkpoints, PRF1 (*p* < 0.01), CTLA4 (p < 0.01), IFNG (*p* < 0.001), CXCL9 (*p* < 0.05), CD274 (p < 0.05), GZMA (p < 0.05), IDO1 (p < 0.01), GZMB (p < 0.01), CD8A (p < 0.01), PDCD1 (p < 0.001) and LAG3 (p < 0.01) were higher in the low-risk group, while only TXB2 (p < 0.01) was higher in the high-risk group. The above results all strongly indicated the potential predictive value of the Riskscore model for ICB therapy efficacy.

To further elucidate the immune spectrum variations between the two risk stratifications, the currently used software with the function of calculating the components of immune cells, including TIMER, XCELL, QUANTISEQ, MCP-counter, EPIC, and CIBERSORT, was applied. The Spearman correlation analysis revealed that immune components are distinctly variable. Cells with antitumor effects, such as CD8+ T cells, CD4+ T cells, B cells and plasma cells, presented negative correlations with the Riskscore, while cells with pro-cancer functions, such as M2 macrophages and cancer-associated fibroblasts, presented positive correlations (Fig. 5C).

Considering all the results above, we further investigated the predictive value of the Riskscore for ICB therapy. The results of IPS analysis for TCGA STAD were downloaded from the TCIA website. PD1 and CTLA4 were enrolled for IPS analysis. Higher IPS represented better accuracy for the more corresponding result (Supplementary Table 5). The results were further classified into 4 groups, namely, ips_ctla4_neg_pd1_neg (CTLA4-negative response and PD1-negative response), ips_ctla4_neg_pd1_pos (CTLA4-negative response and PD1-positive response), ips_ctla4_pos_pd1_neg (CTLA4-positive response and PD1-negative response), and ips_ctla4_pos_pd1_pos (CTLA4-positive response and PD1-positive response). A pairwise comparison of the two risk stratifications in the four groups revealed that the low-risk group was more likely to benefit from treatment with PD1 inhibitors. The average IPS of the low-risk group was higher than that of the high-risk group in both “CTLA4+ PD1+” (Kruskal-Wallis test, *p* = 0.019) and “CTLA4- PD1+” (Kruskal-Wallis test, *p* = 0.013) (Fig. 5D). For the CTLA4 inhibitor group, the average IPS was not significantly different between the two risk groups (Fig. 5D). The results may indicate a better efficacy of this Riskscore model for PD1 therapy response prediction.

### Validation of DEsrlncRNA pair-based signature with Zhongshan cohort

**Fig. 6:**
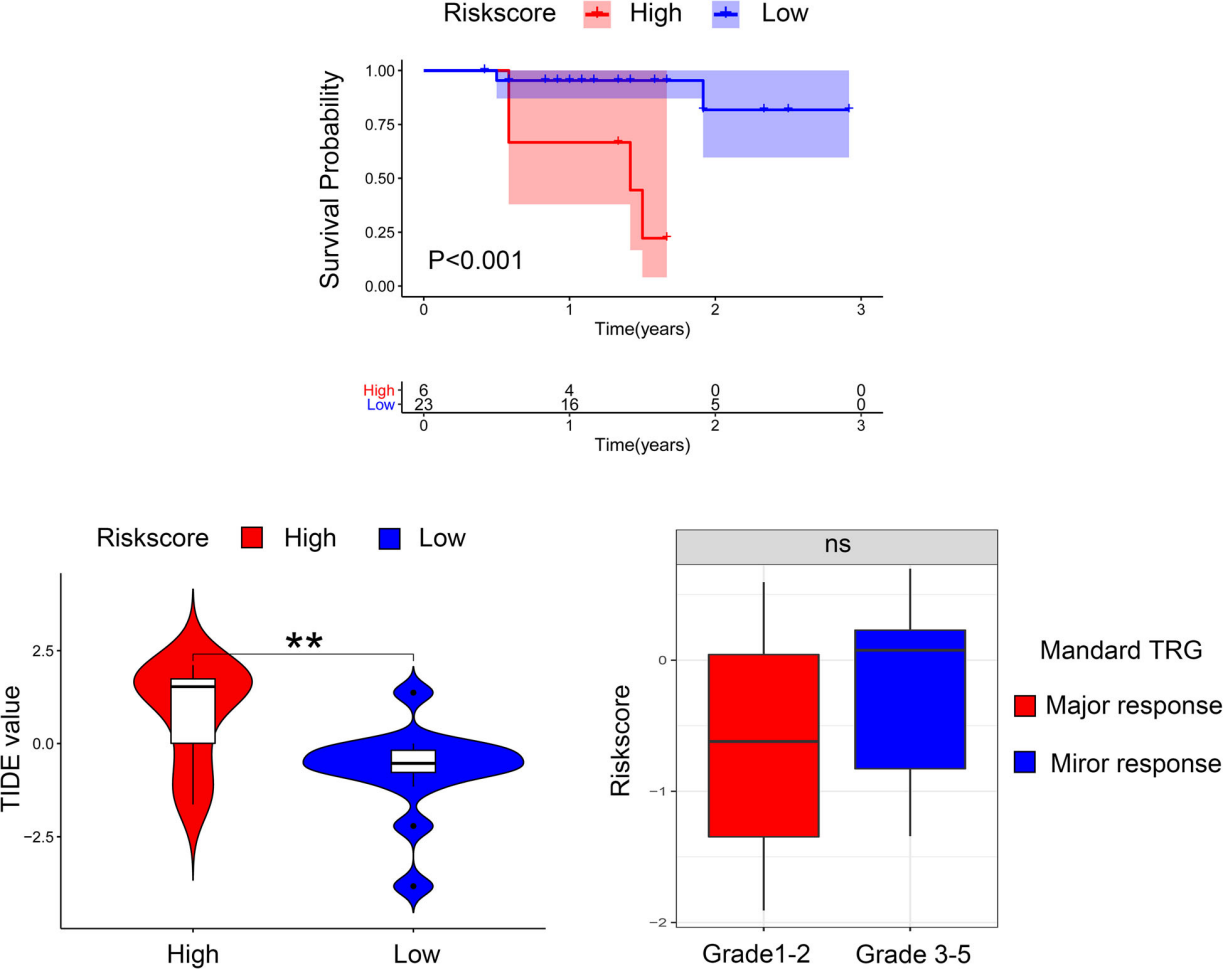
Validation of DEsrlncRNA pair-based signature with Zhongshan cohort. **A** The log rank test shown by Kaplan-Meier curves indicated a better overall survival for low-risk group in Zhongshan cohort (*P* < 0.001) **B** The TIDE value was significantly decreasing in low-risk group, confirming a better response potential to immunotherapy. (Mann-Whitney U test. **p < 0.01). **C** As for chemotherapy response, a lower risk score was also observed in patients diagnose as major response, but the result was not statistically significant. (Kruskal-Wallis test. ns for not significant)

To further validate the application value of DEsrlncRNA pair-based signature, we conduct analysis using Zhongshan cohort. The log rank test shown by Kaplan-Meier curves indicated a better overall survival for low-risk group in Zhongshan cohort based on best cutoff method (*P* < 0.001) (Fig. 6A). The conclusion was highly consistent with our previous finding. To further validate the potential value of this signature for clinical application, we calculated the TIDE value of each sample to make comparisons. The TIDE value was significantly decreasing in low-risk group, confirming a better response potential to immunotherapy (Mann-Whitney U test. **p < 0.01) (Fig. 6B). Since the Zhongshan cohort was composed of patients receiving neoadjuvant, we compared the risk score between major response (Mandard TRG 1–3) and minor response (Mandard TRG 4–5) groups. Despite no statistical significance was observed, the risk score in major response group presented with a decreasing trend (Fig. 6C).

## Discussion

In this study, we aimed to develop a stemness-related lncRNA pair signature with guiding significance for chemotherapy and immunotherapy. The analysis was initiated by collecting stemness-related lncRNAs in GC based on the correlation analysis between stemness genes and lncRNAs. The differentially expressed stemness-related lncRNAs (DEsrlncRNAs) (normal tissues vs tumor tissues) in GC patients from TCGA datasets were further acquired to establish the Riskscore model with Lasso and Cox regression analyses. Since stemness is acknowledged as an important cause of chemotherapy resistance, the Riskscore was tested for its predictive value for chemotherapy response. After that, functional enrichment analysis was conducted. The immune-related pathways were revealed to be highly correlated with lower Riskscore. The results prompted us to explore the relevance between the Riskscore model and immune-related signatures. Immune checkpoint blockade (ICB) therapy-related features, such as microsatellite instability (MSI), tumor mutation burden (TMB) and immune checkpoints, also presented significant correlations with the Riskscore model. We further examined the predictive efficacy of ICB therapy based on immunophenoscore (IPS) analysis. This study could not only provide a stemness-related lncRNA signature for survival prediction in GC patients but also established a model with predictive potentials for GC patients’ sensitivity to chemotherapy and immunotherapy.

With the popularity and development of transcriptome sequencing technology in recent years, GC-related studies focusing on constructing signatures with both coding genes and noncoding RNAs to assist with clinical decision-making are increasing [20, 21] . However, most of these signatures were established based on quantifying the expression levels of transcripts, and the predictive efficacy was more likely to be affected by batch effects. Inspired by the strategy of the immune-related gene pairing model, we tried to develop a reasonable Riskscore signature with two-lncRNA combinations and did not adopt their exact expression levels in the signature. The predictive value of the signature was theocratically more consistent between different RNA-seq data since the batch effect was weakened [16] . Furthermore, this is the first lncRNA pair model established in GC.

Though cancer stemness, lncRNAs and tumor immunity have all emerged as important factors of cancer in recent years, their covariation across cancers has not been systematically elucidated. The first step of our study was to collect stemness-related lncRNAs using coexpression analysis. Screening for differentially expressed lncRNAs between normal and tumor tissues helped further to select candidate stemness-related lncRNAs for signature establishment. After that, we constructed and validated the lncRNA pairs using the method of cyclical single pairing along with a 0-or-1 matrix. The DEsrlncRNA pair-based Riskscore model was finally established. The Riskscore could be a quantitative indicator of stemness. A higher score indicated higher stemness properties. The clinical practicality of the Riskscore was first tested by survival analysis. Patients in the low-risk group experienced better survival based on the Kaplan-Meier test. Further, Cox regression analysis confirmed the Riskscore model as an independent risk factor for GC. The results were as expected since stemness is generally acknowledged as a factor contributing to the malignant characteristics of cancers [15] .

Cancer stem cells (CSCs) have attracted increasing attention for their self-renewal and multipotent properties, as well as their proliferative potential, which gives certain cellular subpopulations the ability to initiate, develop, and progress to cancer [22] . Considering that stemness may lead to chemotherapy resistance, we tested the efficacy of the Riskscore for chemotherapy and targeted therapy responses. Based on the “pRRophetic” package from R software, the IC50 values of 6 GC-concerning chemotherapeutics and targeted drugs (cisplatin, doxorubicin, gemcitabine, vinblastine, pazopanib, and dasatinib) were calculated for each patient. The results revealed that the IC50 values for cytotoxic chemotherapeutics was higher in the high-risk group. Moreover, the IC50 values for targeted drugs was higher in the low-risk group. The results of chemotherapeutics were easy to interpret since the high-risk group harbored stronger stemness properties. The reason why the high-risk group was more likely to benefit from targeted therapy may partially be attributed to the pharmacological actions of these therapies. Targeted drugs may block target proteins that are crucial for the development of stemness properties. For example, hypoxia-inducing factors such as HIFs have been reported to enhance stemness properties in multiple malignancies. Drugs targeting these factors, such as “TH-302”, have been revealed to overcome chemotherapy resistance caused by stemness-related pathways [23]. As contributors to stemness, YAP and TAZ are frequently observed in various cancers and are associated with chemotherapeutic resistance. Dasatinib may inhibit the nuclear localization and target gene expression of YAP and TAZ and thus reverse chemotherapy resistance [24]. However, direct evidence of our hypothesis is still limited.

To further determine the pathways related to the Riskscore model, functional enrichment analysis was performed based on the different risk stratifications. GO enrichment analysis based on the DEGs between the two risk groups revealed that immune-related pathways were highly enriched in the low-risk group. Most of the enriched pathways were found to exert antitumor functions. To validate the results, we further conducted GSEA KEGG analysis. The low-risk group was also enriched in immune-related pathways. To further classify the responsible immune proportions, the currently well-known methods, including TIMER, XCELL, QUANTISEQ, MCP-counter, EPIC, and CIBERSORT, were applied to calculate the proportions of infiltrating immune cells. Cells with antitumor effects, such as CD8+ T cells, CD4+ T cells, B cells and plasma cells, were higher in the low-risk group, while cells with pro-cancer functions, such as M2 macrophages and cancer-associated fibroblasts, were higher in the high-risk group. The results indicated that stemness may lead to a suppressive immune landscape. After reviewing the previous studies, we found that the hypothesis had been partially proven. Alex et al. used gene expression–based metrics to evaluate the association of stemness with immune cell infiltration and genomic, transcriptomic, and clinical parameters across 21 solid cancers. The authors found pervasive negative associations between cancer stemness and anticancer immunity [25]. Ma et al. also proposed the opinion that glioma stem cells (GSCs) and other nontumor cells present in the glioma microenvironment serve as critical regulators of the immune landscape. The accumulation of stem cells is highly correlated with the immunosuppressive microenvironment in glioma [26].

Considering that distinct tumor immunity was observed, we further examined whether the Riskscore was applicable for immunotherapy prediction. Many indexes are regarded as vital criteria for the enrollment of patients suitable for ICB therapy. Of all the indexes, MSI, TMB and the expression levels of immune checkpoints such as PD1 are most important. Higher levels of MSI, TMB and immune checkpoint expression levels have been widely acknowledged as indicators for better ICB therapy response [27]. Our analysis revealed that the Riskscore was negatively correlated with all these indexes. The results may suggest that the low-risk group could better benefit from ICB therapy. To validate this hypothesis, the IPS algorithm was applied to estimate the GC patient response to PD1 and CTLA4 blockade. A lower Riskscore may predict better treatment efficacy for PD1 but not CTLA4.

In recent years, studies committed to constructing stemness-related models to guide clinical decision-making have emerged. Hao et al. conducted a network analysis to collect stemness-related genes in lung cancers. The genes were further processed to establish a signature with predictive value for chemotherapy and immunotherapy responses [28]. Zhang et al. built a 13-mRNA-based prostate cancer stemness model that had high predictive significance for progression-free survival (PFS). The model was also revealed to be closely linked to immune microenvironment changes [29]. Wang et al. established a novel stemness-based classification with appealing implications in discriminating the prognosis and immunotherapy and temozolomide responses of 906 glioblastoma patients. Besides, miRNAs and circRNAs have also been implicated in stemness feature development and related to tumorigenesis. Zhao et al. designed a risk model involving three miRNAs (miR-4521, miR-3682-3p, and miR-1269a). The model could not only served as prognosis predictor, but also being found to be significantly positively or negatively associated with immune infiltration, tumor microenvironment, cancer stemness properties, and tumor mutation burden at different degrees in EC [30]. As a novel circular RNA implicated in cancer development, evaluation of circFAT1 in squamous cell carcinoma (SCC) unifies and regulates the positive association between cancer stemness and immune evasion by promoting STAT3 activation [31]. Interestingly, all the studies mentioned above concluded that stemness features may predict an immunosuppressive landscape. A lower stemness feature was more likely to benefit from ICB therapy.

As for the selection of appropriate patients for ICB therapy, the criteria are still controversial. Of all the reported standards, the Combined Positive Score (CPS) of PD-L1 attracts the most attention. The CPS focuses not only on the percentage of PD-L1-positive cells but also sheds light on the cell types [32]. However, the CPS remains at the standard of manual reading. The efficacy and accuracy of the CPS has actually not been well elucidated [33]. Recently, an inflammatory gene signature was tested for its efficacy to predict the treatment response of ICB treatments for gastric cancer [34]. Considering that the criteria were established based on objective indexes, the detection stability may be better compared with the CPS. Therefore, the Riskscore we established is worthy of further study for its predictive efficacy.

Some limitations in this study inevitably exist. A high predictive efficacy was observed in TCGA STAD datasets and the signature was also validated in Zhongshan cohort. However, we failed to obtain an immunotherapy cohort to validate the practicability of this model. A proper explanation of the non-correlation between the model and AJCC TNM stage is still needed. In addition, all predictive results were derived from bioinformatic methods. A real-world analysis is urgently needed.

## Conclusion

In conclusion, we established a stemness-related lncRNA pair signature for the prediction of GC patient survival. The signature was established based on the comparison of lncRNA expression levels instead of the expression level itself. This method may help overcome the batch effects caused by detection-relevant deviations. The Riskscore was highly correlated with immune pathways. The low-risk group was enriched with antitumor immune-related cells. A lower Riskscore may help predict a better response to ICB therapies in clinical practice.

### Statistical analysis

Statistical analyses were performed utilizing R software 3.6.1, unless specifically noted elsewhere. The details of the specific analysis methods were described in corresponding results.

## Supplementary Information>

**Additional file 1: Supplementary Figure 1 (sFigure 1).** Study flowchart. **Supplementary Figure 2 (sFigure 2)** Stemness-related genes in TCGA-STAD datasets. (A) Comparisons of SRGs between tumor and normal tissues. (Kruskal-Wallis U test. *p < 0.05; **p < 0.01; ***p < 0.001) (B) Univariate Cox regression analysis revealed SRGs with prognosis-predictive value in TCGA cohort(*p* ≤ 0.05). **Supplementary Figure 3 (sFigure 3).** Establishment of the DEsrlncRNA-based signature by the Cox and Lasso regression algorithms. (A and B) Twenty-six out of 57 DEsrlncRNA pairs derived from the prior Cox regression analysis were collected to further conduct Lasso regression analysis. (C) Forest maps show the results of the univariate Cox regression analysis.

**Additional files 2: Supplementary Table 1 (sTable 1).** Stemness related genes. **Supplementary Table 2 (sTable 2).** 240 differentially expressed LncRNAs between 32 normal and 375 tumor RNA-seq data points. **Supplementary Table 3 (sTable 3).** 98 lncRNAs (88 upregulated and 10 downregulated) were identified as DEsrlncRNAs. **Supplementary Table 4 (sTable 4).** 760 differentially expressed genes between the low- and high-risk groups. **Supplementary Table 5 (sTable 5).** The results of IPS analysis for TCGA STAD.

## Data Availability

The datasets generated and/or analyzed during the current study are available in the TCGA STAD repository, [https://portal.gdc.cancer.gov/, December 10, 2020].
