# Supplementary material for: Stemness-related LncRNA pair signature for predicting therapy response in gastric cancer
Source: BMC Cancer. 2021 Sep 29;21:1067. doi: 10.1186/s12885-021-08798-1 (PMC8482617; doi:10.1186/s12885-021-08798-1)
Supplement: Supplementary file 1 — Additional file 1: Supplementary Figure 1 (sFigure 1). Study flowchart. Supplementary Figure 2 (sFigure 2) Stemness-related genes in TCGA-STAD datasets. (A) Comparisons of SRGs between tumor and normal tissues. (Kruskal-Wallis U test. *p < 0.05; **p < 0.01; ***p < 0.001) (B) Univariate Cox regression analysis revealed SRGs with prognosis-predictive value in TCGA cohort(p ≤ 0.05). Supplementary Figure 3 (sFigure 3). Establishment of the DEsrlncRNA-based signature by the Cox and Lasso regression algorithms. (A and B) Twenty-six out of 57 DEsrlncRNA pairs derived from the prior Cox regression analysis were collected to further conduct Lasso regression analysis. (C) Forest maps show the results of the univariate Cox regression analysis. [file 12885_2021_8798_MOESM1_ESM.docx]

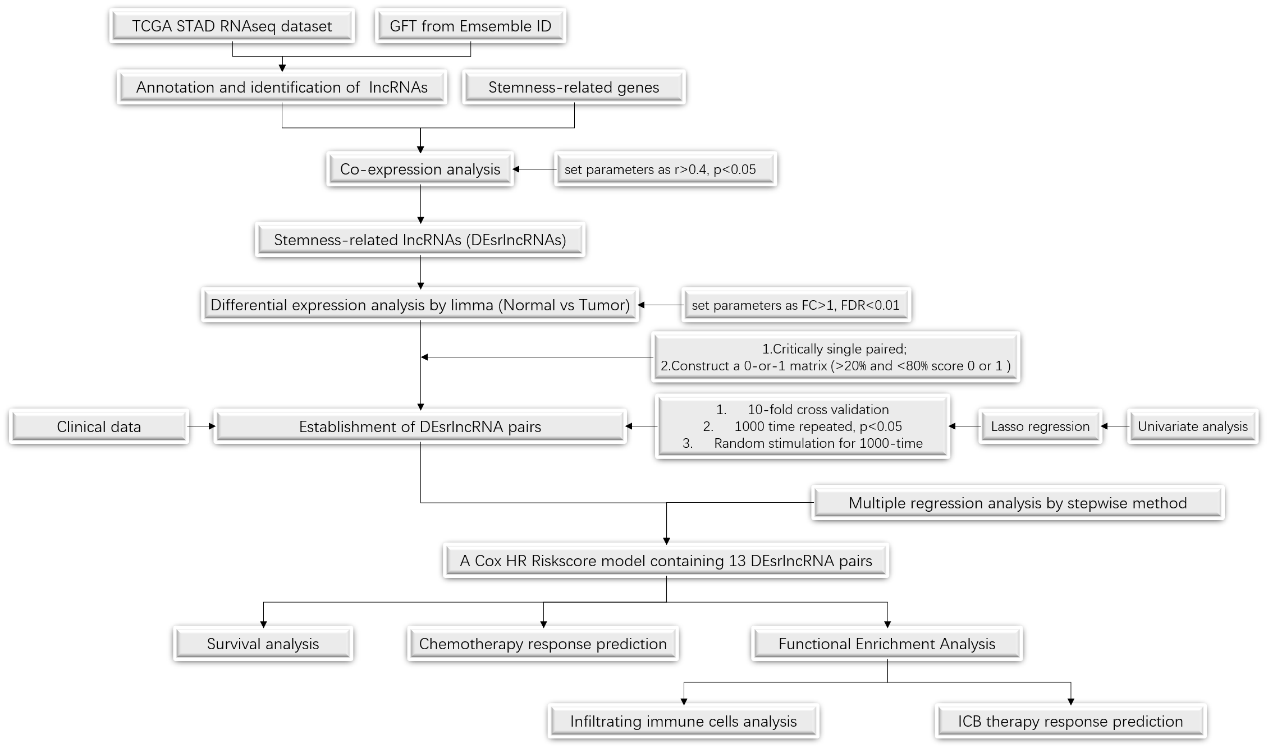


sFigure 1. Study flowchart.


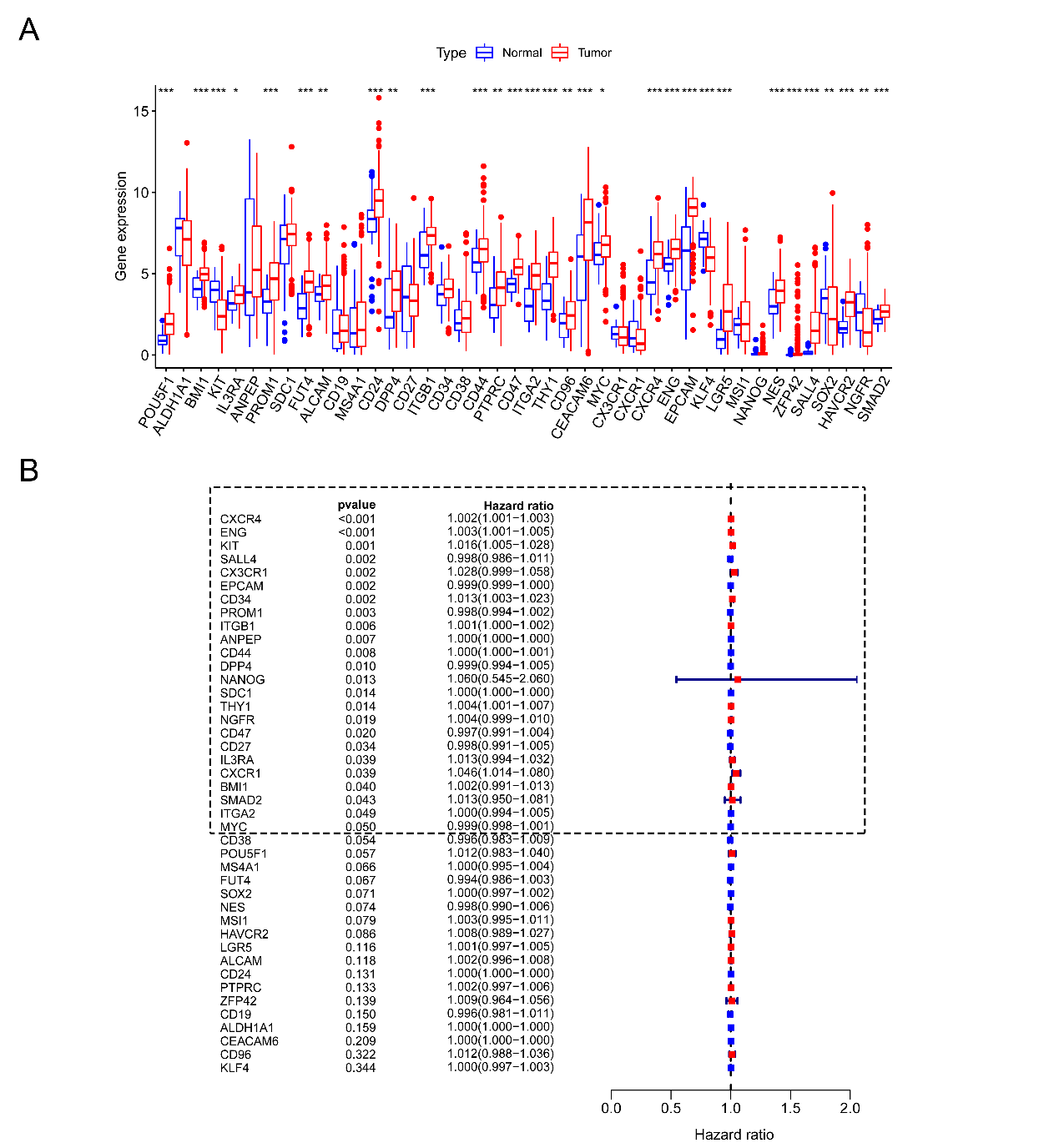
 sFigure 2. Stemness-related genes in TCGA-STAD datasets. (A) Comparisons of SRGs between tumor and normal tissues. (Kruskal-Wallis U test. *p < 0.05; **p < 0.01; ***p < 0.001) (B) Univariate Cox regression analysis revealed SRGs with prognosis-predictive value in TCGA cohort(p≤0.05).


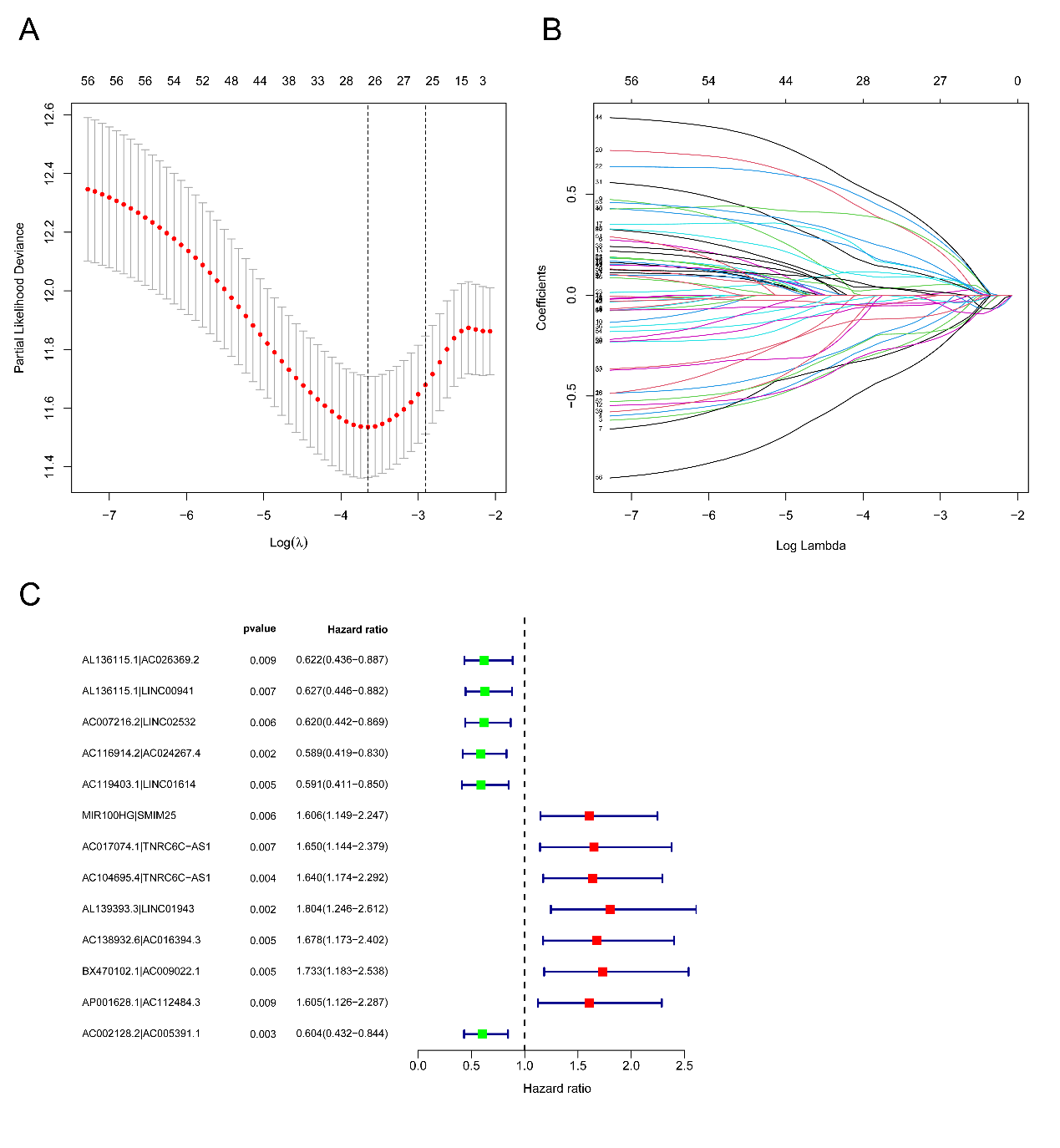


sFigure 3. Establishment of the DEsrlncRNA-based signature by the Cox and Lasso regression algorithms. (A and B) Twenty-six out of 57 DEsrlncRNA pairs derived from the prior Cox regression analysis were collected to further conduct Lasso regression analysis. (C) Forest maps show the results of the univariate Cox regression analysis.
